# Experimental and Numerical Study of Printing Strategy Impact on the Mechanical Properties of Sustainable PLA Materials

**DOI:** 10.3390/polym15244639

**Published:** 2023-12-07

**Authors:** Emil Spišák, Ema Nováková-Marcinčínová, Janka Majerníková, Peter Mulidrán, Ľudmila Nováková-Marcinčínová

**Affiliations:** 1 Faculty of Mechanical Engineering, Technical University of Košice, Letná 9, 042 00 Košice, Slovakia; ema.novakova-marcincinova@tuke.sk (E.N.-M.); janka.majernikova@tuke.sk (J.M.); peter.mulidran@tuke.sk (P.M.); 2 Faculty of Manufacturing Technologies with a Seat in Prešov, Technical University of Kosice, Bayerova 1, 080 01 Prešov, Slovakia; ludmila.novakovamarcincinova@tuke.sk

**Keywords:** wood PLA, PLA material, 3D printing strategy, fused deposition modelling, numerical simulation

## Abstract

This article is focused on a mechanical properties investigation of three types of sustainable poly lactic acid materials manufactured using the fused filament fabrication process. The purpose of this work was to study the impact of printing strategies on the mechanical properties and predict mechanical behavior under tensile loading using finite element analysis. The testing of mechanical properties was conducted according to the ISO 527 standard. The numerical simulations were conducted in Simufact Forming 2022 software. Analysis of the experimental data showed a dependance of mechanical properties on the used printing strategy. The Clear PLA samples printed in the XY plane exhibited a 43% reduction in tensile strength and a 49% reduction in elongation compared to samples printed from the same material in YZ plane. The experimental results show the influence of the printing orientation on the mechanical properties of 3D-printed samples.

## 1. Introduction

In recent years, research on different types of poly lactic acid (PLA) materials has increased because of their possible use in industrial applications. Today, the research is mainly conducted on fiber-reinforced composites with a polymer matrix. The fibers are usually made of synthetic materials, such as carbon or glass, because of their positive impact on the strength properties of 3D-printed samples [1,2]. PLA, in combination with reinforcing or additives can be used to produce durable and functional parts that can be used in engineering applications [3,4,5].

Natural materials, such as wood, flax, coffee, and wheat, are increasingly being incorporated into 3D-printing filaments as additives or reinforcements. This trend is driven by several factors, including the biodegradability, lightweight nature, and low cost of these materials, as well as the potential enhancement of the mechanical properties they can offer. Wood-polymer filaments are gaining attention due to their potential applications in art, construction, and industrial manufacturing [6,7,8].

Fused filament fabrication (FFF) technology shows great capability in printing natural fiber-reinforced composites with a PLA matrix, making them more desirable than other materials. These types of composites are recently being used by the biomedical [9], furniture [8], packaging [10], and naval industries [11]. The automotive sector is starting to use natural-fiber composites with polymer matrix for the production of door panels, door and dashboard trims, and seat backs [12]. Despite the promising potential of FFF-produced parts, it is crucial to thoroughly understand their mechanical properties before adopting them for widespread industrial applications. Therefore, research is essential to elucidate the influence of natural additives and fibers on the mechanical behavior of these materials.

Several studies have investigated the mechanical properties of 3D-printed materials, with a particular focus on their behavior under tensile loading. Le Duigou et al. [13] conducted research regarding flax fiber orientation and their impact on mechanical properties on printed samples. Their results suggest that samples with fibers oriented in the x-axis exhibited better values of mechanical properties. Kariz et al.’s [14] paper focused on investigating of mechanical properties of composite PLA-wood samples. The addition of wood particles decreased strength of 3D-printed samples because of increased numbers of pores in the structure. Tao et al. [15] published a paper in which they investigated the properties of wood flour filled PLA composite. Their results suggest that by introduction of wood flour to the PLA matrix the initial deformation resistance can be improved. Spišák et al. [16] investigated the impact of the printing strategy on the mechanical behavior of five types of PLA composites. Their results suggest that parts extruded in length orientation exhibit higher tensile strength, especially carbon-PLA composites. Hsueh et al.’s [5] work evaluated the effect of printing temperature and speed on the mechanical properties of PLA and Polyethylene terephthalate glycol (PETG) using tensile, compression, and bending tests. The PLA samples printed at higher temperature show greater values of tensile strength. Guessasma et al. [17] studied the effect of printing temperature on the mechanical properties of wood-PLA material. They suggest that the optimal printing temperature is 210 °C for this type of material. The work from Živčák et al. [18] studied the impact of the heat treatment process on mechanical properties of 3D-printed samples made of titanium alloy Ti6Al4. The use of age-hardening increased tensile strength after 3D-printed samples were made.

Numerical simulations are frequently employed to predict the mechanical behavior of metals and polymers [19,20,21]. However, numerical predictions of the mechanical behavior of natural fiber composites with polymer matrix produced by FFF technology are still in the early stages of research. Ezzaraa et al. [22] conducted numerical and experimental analyses of 3D-printed samples made of a PLA-wood composite. The impact of the wood volume fraction and internal porosity on mechanical properties was investigated. The relative error of numerical predictions did not exceed 10%. The work of Dutra et al. [23] focused on predicting the mechanical behavior of nylon matrix-based composite materials. They stated that Young’s modulus values have a great impact on numerical prediction accuracy. Park and Rosen’s [24] paper focused on the prediction of mechanical properties of 3D-printed samples. They suggest that FFF process parameters and internal voids affect Young’s modulus. Polyzos et al. [25] proposed a numerical multi-scale method to calculate the elastic properties of 3D-printed materials reinforced with continuous fibers. The results obtained using the ABAQUS 2021 software were validated against analytical models and experimental data, demonstrating a high degree of agreement. Chen et al. [26] proposed a damage model to calculate the mechanical behavior of 3D-printed origami shaped tubes under uniaxial compression. Their numerical results indicate a good agreement with experimental results. The work of Monaldo et al. [27] focused on the investigation of mechanical properties of 3D-printed samples made of PLA material. Tensile and three-point bending tests were carried out. Numerical predictions of mechanical behavior were conducted using a new model based on the first-shear deformation theory. The experimental results were compared with numerical ones, analysis suggesting the effectiveness of the proposed modelling approach.

Based on the literature review, it is evident that the investigation of the mechanical properties of 3D-printed parts made of PLA and natural fiber PLA composites is a topic of considerable interest among researchers. This work aims to contribute to this field by exploring the mechanical properties of three types of PLA filaments—clear PLA filament, colored PLA filament, and composite PLA-wood filament—under tensile loading. The impact of the printing strategy on their mechanical behavior will also be analyzed. Additionally, numerical simulations using the Simufact Forming 2022 software will be conducted to predict the mechanical properties of the three types of PLA filaments under uniaxial tension, this software is usually used for predicting metal forming processes, in this work it was used to predict the mechanical behavior of PLA materials. This study is expected to provide valuable insights into the mechanical behavior of these materials under tensile loading.

## 2. Materials and Methods

### 2.1. Materials

Three types of PLA filaments were used in this experiment to study the impact of printing strategies on mechanical properties. Translucent (Clear PLA), colored PLA (White PLA), and composite PLA with wood saw particles (Wood PLA) were used for testing. Three types of PLA filaments were obtained from the same manufacturer Fillamentum Manufacturing Czech s.r.o. (Hulín, Czechia).

PLA plastic differs from plastics made of petroleum products. Moreover, it is made from renewable sources. Usually they do not contain any chemicals or additives, such as common petroleum plastics. They save on the consumption of non-renewable fossil resources, such as crude oil. They reduce greenhouse gas production, such as CO_2_ [28]. They comply with European and world standards and regulations for health and food safety [13,29]. Bioaugmentation and biostimulating techniques are important approaches to increasing hydrolysis and the activity of PLA-degrading bacteria to accelerate PLA biodegradation. Bioaugmentation refers to the addition of a bacterium to a medium where an indigenous population of bacteria occurs. This technique could accelerate the biodegradation of PLA in the medium by adding a specific PLA-degrading bacterium [14,30]. WPC material is composed of wooden particles and polymers. Products made from wood plastic copolymer (WPC) look like wood, but they do not have disadvantageous properties such as mustiness, color liability to change, or possible damage caused by weather influence. Water absorption is more pronounced in WFCs with a hydrophilic matrix, such as PLA, and leads to decreased mechanical stiffness and strength [31]. The quantities of natural fiber-reinforced plastic composites produced in the European Union in 2017 were only 0.41 million tons, and with annual growth rates of 3%, their application in consumer goods and building products is comparatively low [32]. Properties of Clear PLA, White PLA and Wood PLA are shown in Table 1.

### 2.2. Test Samples Production Using RepRap3D Printer

**Figure 1:**
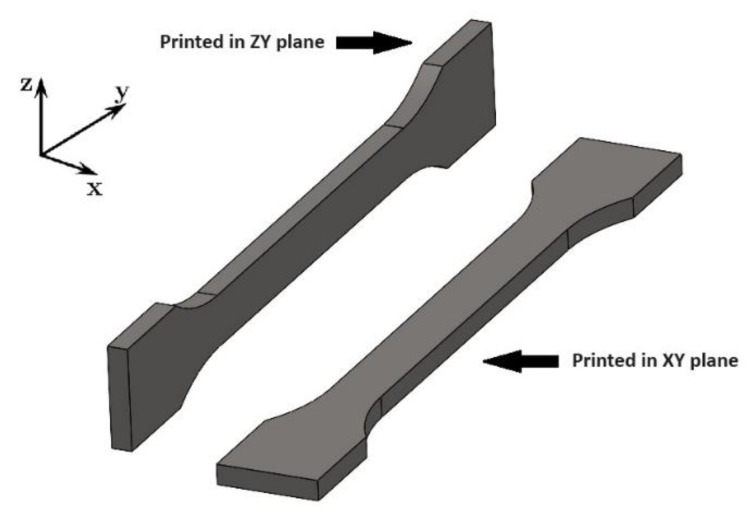
Printing orientation of tensile test specimens.

A CAD model of uniaxial sample intended for 3D-printing was designed in Solidworks 2022 software (Dassualt Systems, Waltham, MA, USA). Subsequently, the test specimens had to be converted to STL format. The Repetier-Host 2.1 software (Hot-World GmbH & Co. KG, Willich, Germany) for editing and preparation of 3D models for 3D-printing was used to slice the model into layers and the desired printing speed and strategy was chosen. This software also allows displaying individual layers of sample intended for printing. Three types of PLA filaments were used for printing, Clear PLA, White PLA and composite Wood PLA. These filaments were produced by the same manufacturer. The printing temperature was 170 °C for Wood PLA. Clear PLA and White PLA materials were printed at 190 °C. Printing speed was 60 mm/s for each type of filament. The layer height was 0.4 mm for all samples produced. Tensile test samples printed in the YZ plane consisted of 52 layers. Samples printed in the XY plane consisted of 10 layers. Printing orientations and planes are displayed in Figure 1.

**Figure 2:**
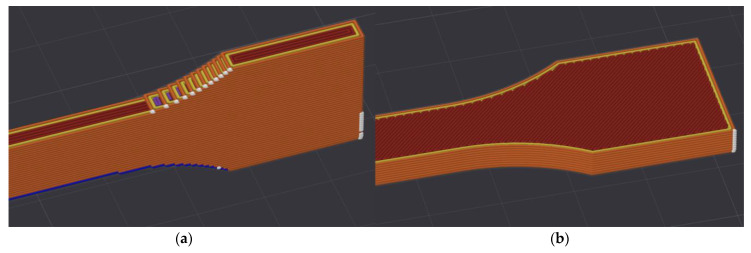
Printing strategies (**a**) linear strategy used in YZ plane (**b**) zig-zag strategy used in XY plane.

Two types of printing strategies—linear and zig-zag—used for the production of samples are shown in Figure 2. The zig-zag strategy used in the XY plane consisted of diagonal fibers under a 45° angle in one layer, the next layer also consisted of diagonal fibers with a 45° orientation but mirrored along length of the sample. Two outer layers and the boundary of sample was printed using linear fibers. The samples were produced with 100% infill.

### 2.3. Uniaxial Tensile Test of 3D-Printed Samples Made of Different Types of PLA Material 

**Figure 3:**
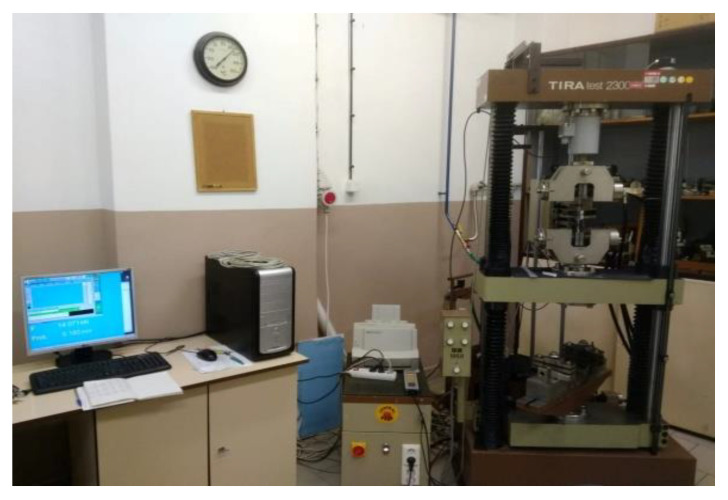
TiraTest 2300 testing device.

Experimental testing of mechanical properties on 3D-printed PLA samples were realized according to the STN ISO 527 standard [33,34]. The testing device, TIRA-test 2300 (TIRA Maschinenbau GmbH, Rauenstein, Germany), in Figure 3 was used in the Laboratory of Mechanical and Technological Testing at the Institute of Technology and Material Engineering and at the Faculty of Mechanical Engineering of the Technical University in Košice. The testing machine can be used for mechanical properties measuring of various materials such as paper, plastics, rubber, textile, metals, etc. It can measure the tensile strength, compressive strength, and the bending strength. It also provides high user comfort for testing procedure-controlling evaluation of measured data and statistical options followed by archival service. The software platform works under the Windows operation system with full localization services. This universal testing device, TIRA-test 2300, is computer-controlled testing machine, mainly used for tensile tests with a maximal testing force of 100 kN and a load range of 1, 10 and 100 kN based on the used tensometer equipment. The machine can be also used for welded and glued joints strength testing [20,35].

**Figure 4:**
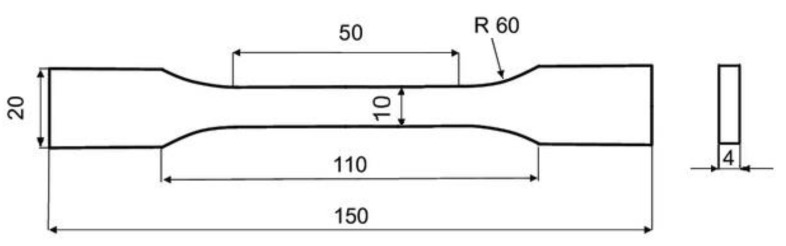
Tensile test specimen dimensions according to ISO 527 [mm].

**Figure 5:**
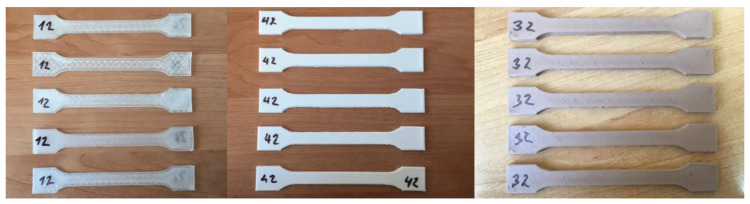
Samples made from Clear PLA, White PLA and Wood PLA printed in XY plane.

In total, 30 printed samples were tested, measured, and evaluated. Dimensions of test samples are shown in Figure 4. From which, 10 samples were made from clear PLA plastic; 10 samples were made of white PLA plastic; and 10 samples were made of PLA plastic with an admixture of wood sawdust. Each type of tested PLA was printed in the YZ and XY plane, 5 samples for each printing plane strategy. Figure 5 shows samples made of three types of PLA material printed in the XY plane. The test specimens were under tensile loading until rupture. The test speed was set at 10 mm/min on the test machine. Testing results were recorded in the computer software and then evaluated automatically. Based on the measured force values, the tensile strength σ_M_ [MPa] was determined, as well as the elongation at the tensile strength ε_M_ [%].

### 2.4. Simulation of Uniaxial Tensile Test Using Simufact Forming Software 

**Figure 6:**
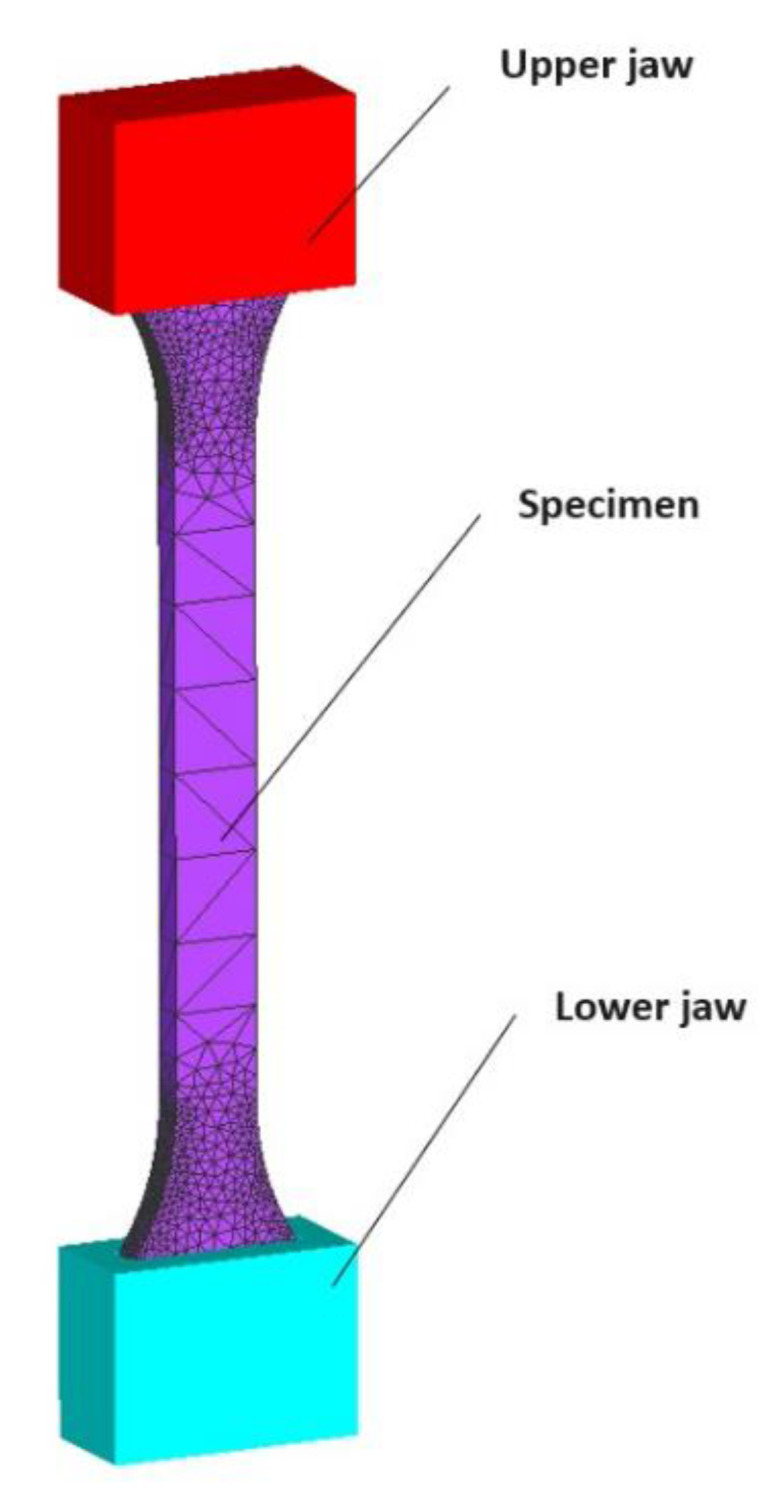
Tensile test model used in numerical simulation.

Tensile test simulation was performed using the Simufact Forming 2022 software (Simufact Engineering GmBH, Hamburg, Germany). This mentioned software is widely used for sheet-metal forming simulations. The CAD model, representing the tensile test shown in Figure 6, was imported into the software. The upper and lower jaw were defined as rigid dies. The upper jaw moved in one axis to simulate the tensile test, thus, applying tensile loading on the part. The specimen was fixed in the jaws. The tensile test specimen was mashed with square shell elements, and the size of the element was 1.5 mm. The material properties of three types of PLA materials, such as tensile strength and elongation at break were defined based on the experimental tensile test results of samples made in the YZ plane. The Hollomon isotropic hardening law was used to define the hardening of the material. Isotropic hardening rules describe yield surface expansion when stress is applied to the material, but the shape of the yield surface does not change. The Cockroft-Latham damage model was used to predict fracture.

## 3. Results

### 3.1. Uniaxial Tensile Test Results 

The measured and calculated values obtained from the tensile test of the printed samples are listed for individual PLA test samples in the following tables (Table 2, Table 3 and Table 4).

**Figure 7:**
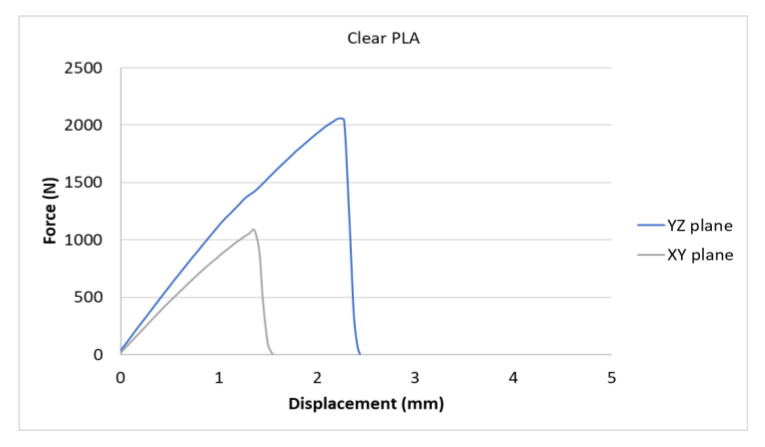
Diagram of tensile test of Clear PLA printed in YZ and XY planes.

**Figure 8:**
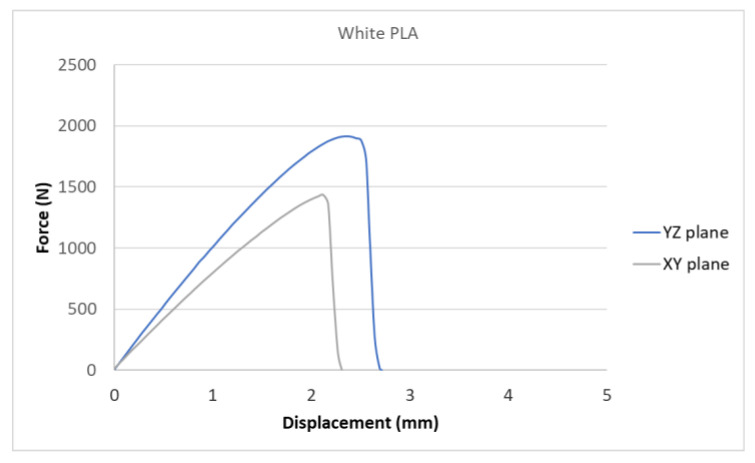
Diagram of tensile test of White PLA printed in YZ and XY planes.

**Figure 9:**
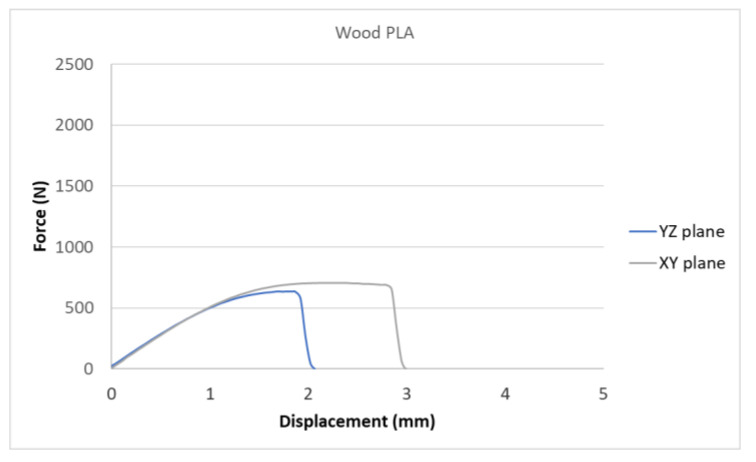
Diagram of tensile test of composite PLA-wood printed in YZ and XY planes.

**Figure 10:**
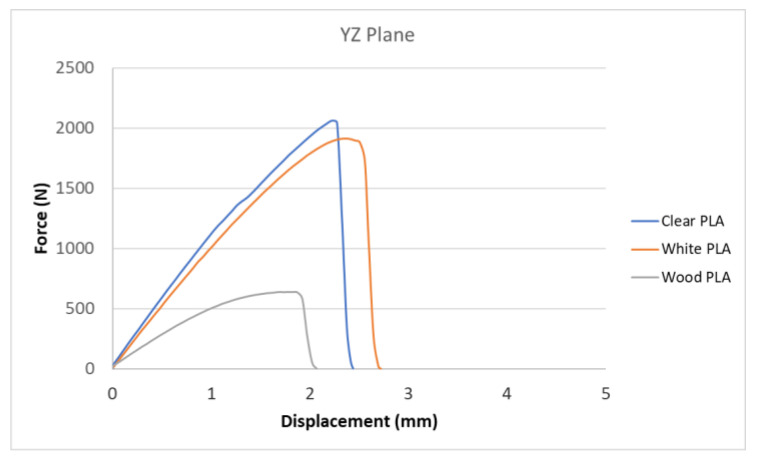
Comparison of tensile test curves for three types of PLA printed in YZ plane.

**Figure 11:**
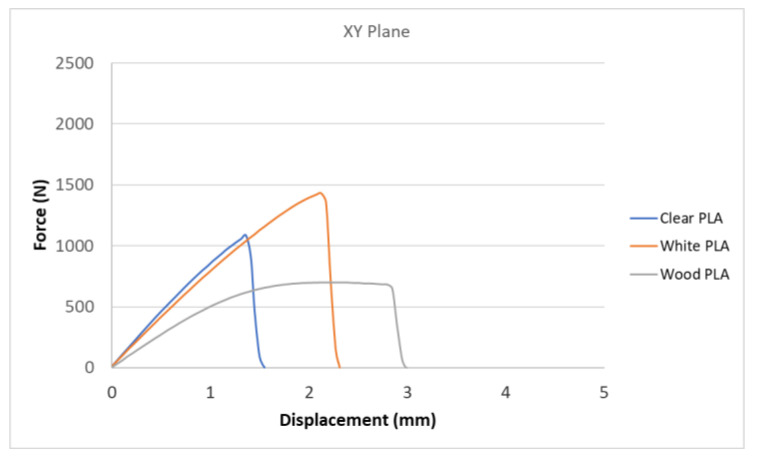
Comparison of tensile test curves for three types of PLA printed in XY plane.

Figure 7, Figure 8, Figure 9, Figure 10 and Figure 11 show the dependence of the displacement (mm) on the loading force F (N), which was evaluated using the data from the testing machine software during the uniaxial tensile test.

**Figure 12:**
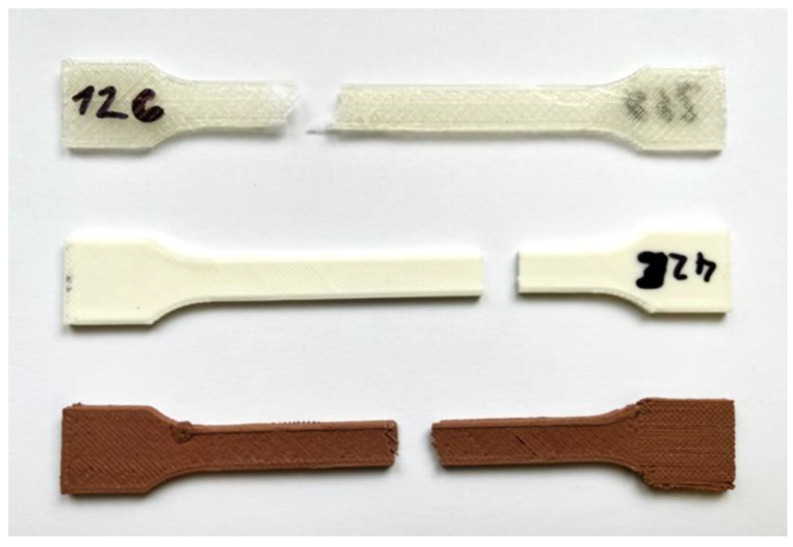
Samples made of clear PLA, white PLA, and PLA-wood composite printed in XY plane.

Tested specimens after the implementation of the static tensile test produced by the FDM method on a Rep-Rap 3D printer (eMotion Tech, Toulouse, France) from individual PLA plastics printed in the XY plane (Figure 12).

### 3.2. Tensile Strength Evaluation of Individual Tensile Test Samples

**Figure 13:**
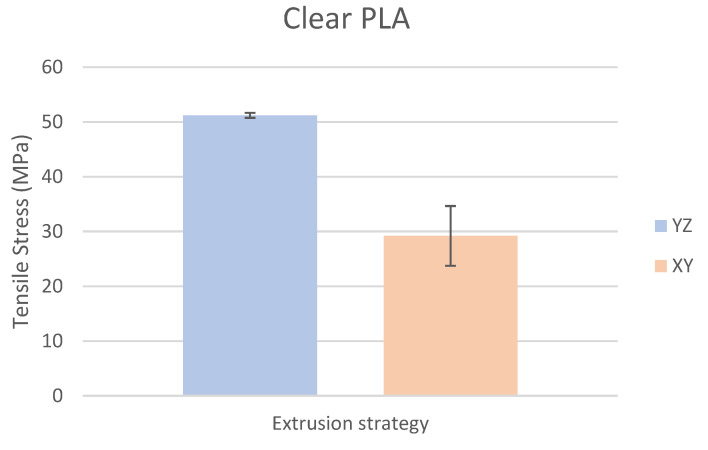
Comparison of tensile stress σ_M_ of Clear PLA printed in the YZ and XY planes.

**Figure 14:**
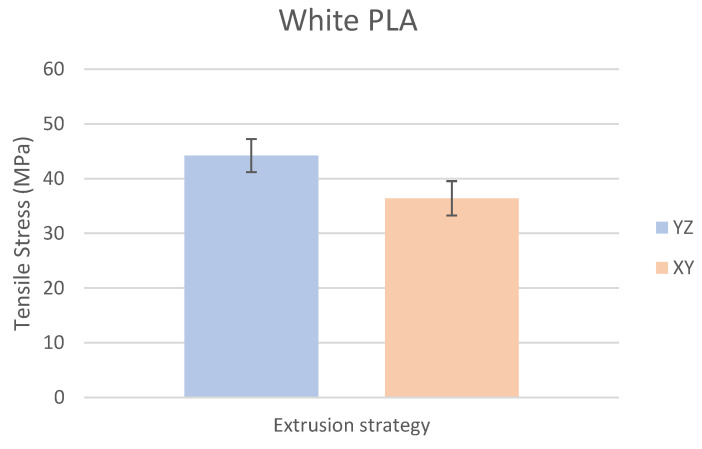
Comparison of tensile stress σ_M_ of White PLA printed in YZ and XY planes.

**Figure 15:**
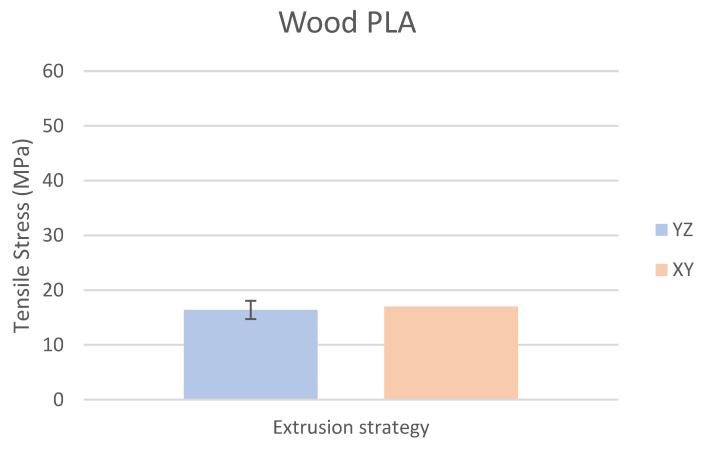
Comparison of tensile stress σ_M_ of Wood PLA s printed in YZ and XY planes.

Based on the performed experiments and measured values of the tensile strength values σ_M_ [MPa] for different types of PLA—clear, white and Wood PLA mixture—using two different printing strategies, the following planes can be stated. The tensile strength values σ_M_ of samples made of Clear PLA plastic (Figure 13) and specimens produced in YZ plane had the tensile strength σ_M_ of 51.2 MPa. The samples printed in the XY plane from the same material and the Clear PLA showed lower values of tensile strength compared to samples printed in the YZ plane, the difference is approximately 25%. The overall strength values of White PLA samples (Figure 14) were lower compared to samples made of clear PLA. Samples made of Wood PLA material (Figure 15) showed almost the same average value of tensile strength for both extrusion strategies, the difference in tensile strength between samples printed in the YZ and XY plane was less than 1 MPa. The lowest values of tensile strength were measured for samples made of composite PLA-wood.

**Figure 16:**
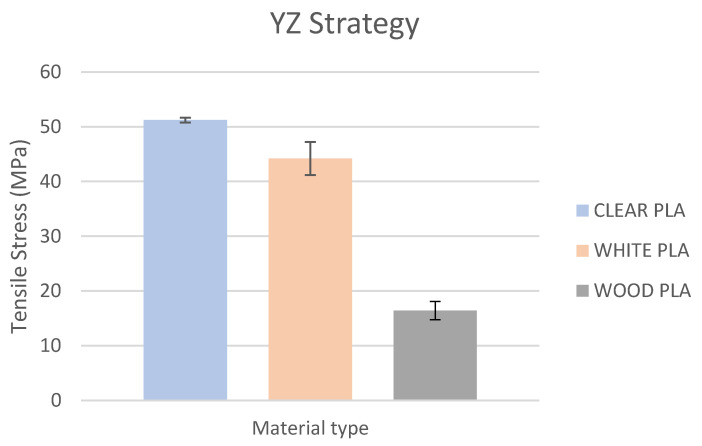
Comparison of filaments tensile strength printed in the YZ plane.

**Figure 17:**
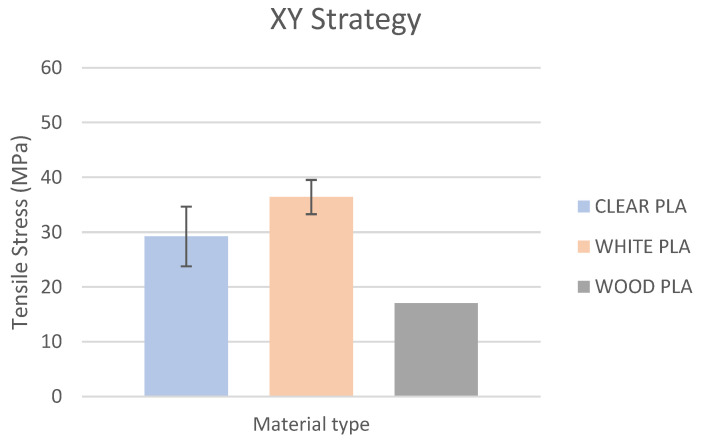
Comparison of filaments tensile printed in the XY plane.

The clear PLA material shows the highest tensile strength of 51.2 MPa and, therefore, can be found to be the strongest when printed in the YZ plane (Figure 13). The tensile strength of White PLA and PLA-wood is significantly lower. The tensile strength values of White PLA had the value of tensile strength of 36.4 MPa when printed in XY plane and 44.2 MPA when printed in YZ plane. The samples made of composite PLA-Wood material showed the lowest values of strength of all tested material for both printing strategies, in both printing planes. PLA-Wood material did not exhibit strong impact of printing, and extrusion strategy on the tensile strength results. A comparison of all materials tensile strength for each strategy are shown in Figure 16 and Figure 17.

### 3.3. Microstructure and Fracture Evaluation

**Figure 18:**
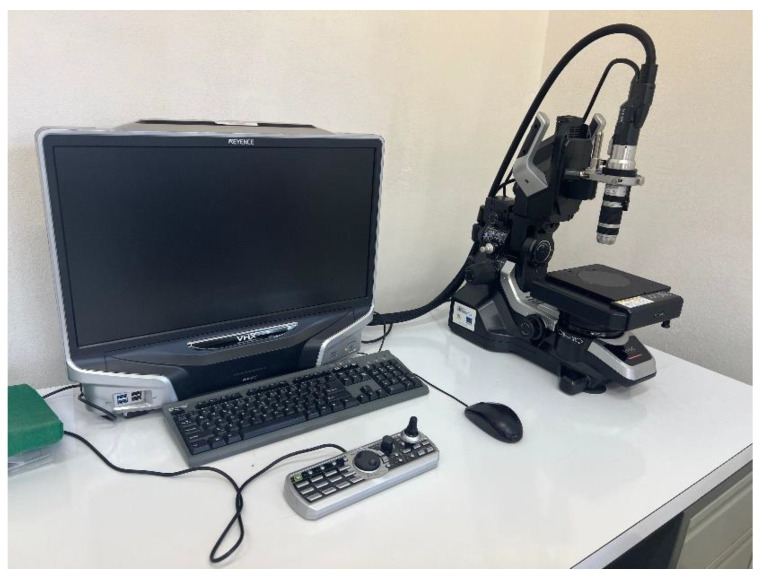
Digital light microscope Keyence VHX-500.

The samples were studied at the Faculty of Mechanical Engineering, Technical University of Košice in the Metallography laboratory, under constant ambient conditions using a Keyence VHX—500 digital microscope—(Keyence SV, Mechelen, Belgium). This microscope has a resolution of 18 million pixels. It is shown in Figure 18.

**Figure 19:**
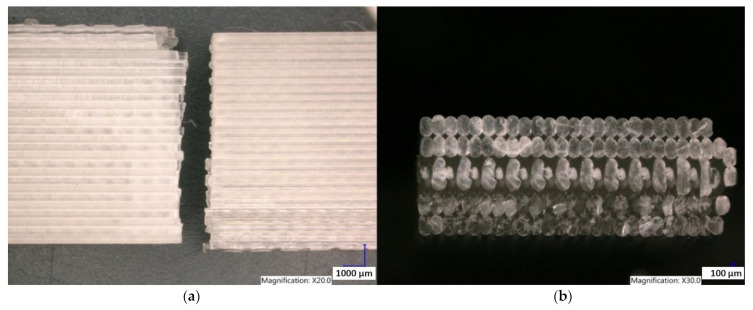
Structure of Clear PLA printed in the YZ plane (**a**) sample after the tensile test (**b**) fracture surface.

**Figure 20:**
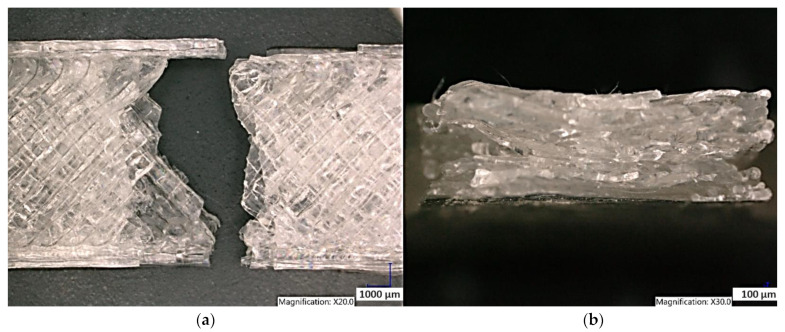
Structure of the Clear PLA printed in the XY plane (**a**) sample after the tensile test (**b**) fracture surface.

Observation of the Clear PLA sample structure printed in the YZ plane shows the failure of the sample due to the brittle fracture of the individual fibers perpendicular to previously applied tensile load in all layers. It is also possible to observe the individual fibers of the samples and the interconnections of the individual layers when printing the samples in the YZ plane (Figure 19). The structure of the Clear PLA sample printed in the XY plane is shown in Figure 20. The fracture shape is not the same as the fracture of sample made in the YZ plane.

**Figure 21:**
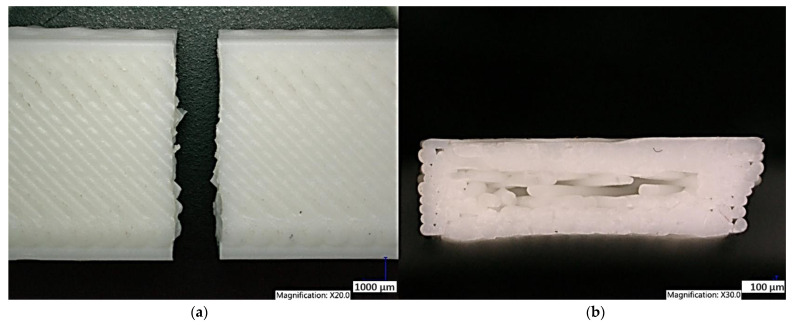
Structure of the White PLA printed in the XY plane (**a**) sample after the tensile test (**b**) fracture surface.

**Figure 22:**
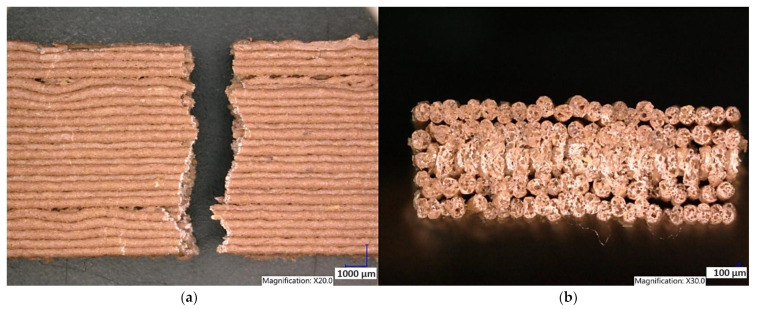
Structure of the Wood PLA printed in the YZ plane (**a**) sample after the tensile test (**b**) fracture surface.

White PLA material structure printed in the YZ plane is shown in Figure 21. The brittle fracture is observed perpendicular to the previously applied tensile load. The PLA-wood composite structure printed in the YZ plane is shown in Figure 22. The fracture surface shows high porosity of the PLA-wood material. This type of material showed lowest values of tensile strength.

### 3.4. Numerical Simulation Results of Tensile Test

**Figure 23:**
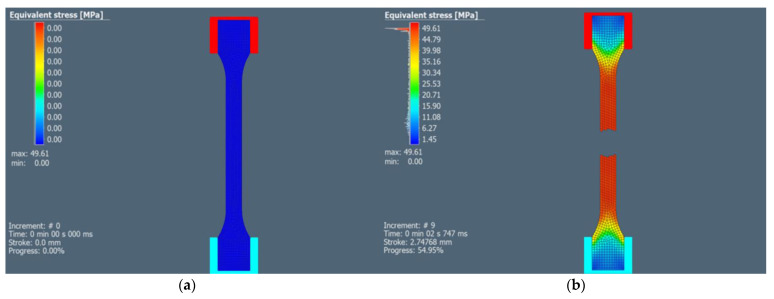
Numerical simulation of the Clear PLA tensile test (**a**) sample before tensile test (**b**) sample after tensile test.

Numerical stress results σ_ms_ show minimal deviations from the average experimental stress results of samples printed in the YZ plane extrusion strategy σ_ma_. The standard deviations of stress σ_mdev_ and elongation ε_mdev_ experimental results are shown in Table 5. The comparison of experimental and simulation results is also shown in Table 5. Stress results of the Clear PLA material after tensile test using numerical simulation are shown in Figure 23.

The elongation values of the tensile test sample were higher in numerical simulations ε_ms_ compared to experimental ones ε_ma_. The deviations of predicted stress values from the experimental results were calculated according to the formula:

(1) $$Dev\;X=\frac{X_{sim}-X_{exp}}{X_{exp}}\times 100\;[\%]$$

where *Dev X* is a deviation of predicted stress value σ_ms_ from the average value of stress σ_ma_, *X_sim_* is the predicted stress value, and *X_exp_* is the average experimental stress value.

## 4. Discussion

Previous works in the field of testing PLA-based materials are usually oriented on the composite materials with PLA matrix and synthetic reinforcement, such as PLA-GF and PLA-CF [16,36,37]. More recently, research is oriented on the PLA materials with natural reinforcement (wood, wheat, bamboo, coffee, etc., particles) which are better for environment [8,9,38]. This work was oriented on the determination of mechanical properties of PLA based 3D-printed samples under uniaxial tensile loading. Three types of filaments were used in the research—Clear PLA, White PLA, and composite Wood PLA material. Two types of extrusion strategy were used to produce tensile test samples to test the impact of printing strategy. The comparison of experimental results with deviations is presented in Table 6. 

Experimental results show that the Clear PLA material was the strongest material of three types of materials tested. The average tensile strength of this material was 51.2 MPa. The White PLA material showed lower values of tensile strength compared to the Clear PLA. This can be attributed to the additives which changed the color of the filament to the white one. These additives can increase the micro porosity of the material and reduce the adhesion of layers, thus reducing the tensile strength [39]. The composite material Wood PLA exhibited the lowest tensile strength and lowest elongation. The reason for this can be attributed to pores which are present in each layer of prepared samples, they can be easily observed on the fracture surface in Figure 22b. These pores were also observed by the work presented in [40,41]. The printing strategy had a significant impact on the samples tensile strength made of Clear PLA and White PLA. The tensile strength reduction of 43% was observed for the Clear PLA samples printed in the XY plane compared to samples printed in the YZ plane. Likewise, the reduction of tensile strength was observed in the samples made of White PLA printed in the XY plane. The reduction in tensile strength was 18% compared to samples printed in YZ plane. The use of zig-zag strategy (printing in XY plane) also had negative impact on the elongation of the samples made of the Clear PLA and White PLA. The average elongation of Clear PLA samples printed in the XY plane was 49% lower compared to samples printed in the YZ plane. For samples made of the White PLA material the elongation reduction was only marginal when using zig-zag strategy (printing in XY plane). The brittle fracture of the Clear PLA and White PLA can be attributed to their low elongation. PLA is amorphous glassy polymer to semi-crystalline and highly crystalline polymer. Glassy polymers are rigid and hard, lacking the flexibility and elasticity seen in rubbery polymers. The polymer chains are immobilized in a densely packed, non-crystalline structure, limiting their movement. Glassy polymers tend to be relatively brittle, meaning they are prone to fracture or failure under stress without significant deformation [42,43]. The extrusion strategy had almost no impact on the tensile strength of the composite Wood PLA samples. The elongation was greater for samples printed in the XY plane. The difference between both strategies is 11%. The higher tensile stress values for the Clear PLA and White PLA samples printed in the YZ plane can be probably attributed to the orientation of fibers in each layer which were parallel to the applied force. The numerical results of tensile stress and elongation were compared with experimental ones as shown in Table 5. The numerical results were close to the experimental ones. The deviations of predicted tensile stress from experimental tensile stress values were—3.12% for Clear PLA, 0.90% for White PLA and 1.21% for Wood PLA.

## 5. Conclusions

The use of sustainable composite PLA materials with natural particles or fibers to produce real parts, jigs and fixtures is on the rise, thus, these types of materials must be properly studied. In this research, three types of PLA materials—Clear PLA, White PLA and PLA—mixed with wood particles were tested and evaluated. It was determined which type of PLA samples were the strongest. The structure of extruded samples after tensile tests were observed and evaluated. Furthermore, numerical simulations of tensile test of the three types of PLA were conducted, and numerical results were compared with experimental ones. Future research might include investigation of other types of sustainable natural-based particles, PLA composites, and different types of printing strategies, and their impact on mechanical properties. Based on the evaluation and analysis of the experimental and numerical results, it can be stated that:

- The type of PLA filament has a significant impact on the mechanical properties and deformation behavior of 3D-printed samples. The lowest value of tensile strength 16.4 MPA was observed on samples made of the composite PLA-wood filament, the highest strength value of 51.2 MPa was observed for the Clear PLA.
- The printing plane strategy has a significant impact on the mechanical properties of the printed PLA based material. The Clear PLA samples printed in the XY plane had a 43% reduction in strength, compared to samples printed in the YZ plane.
- The printing strategy had a minimal impact on the tensile strength of the Wood PLA, the difference between samples printed in the YZ and XY plane is less than 6%.
- All types of PLA showed very low values of elongation. Clear PLA and White PLA materials showed brittle fractures during the tensile test.
- Composite PLA-wood showed some plastic deformation, but overall, elongation of 3.6% and the strength of 16.4 MPa were the lowest, especially for samples printed in the YZ plane.
- Simufact Forming software can be used to predict deformation behavior and tensile strength of the PLA based samples. The maximum deviation −3.12% of predicted tensile strength from measured tensile strength was observed for the Clear PLA material.

## Figures and Tables

**Table 1:** Properties of tested PLA materials provided by manufacturer.

Material	Material Density	Tensile Modulus	Tensile Strength	Elongation at Break
	[g/cm^3^]	[MPa]	[MPa]	[%]
Clear PLA	1.24	3500	50	≤5
White PLA	1.24	3500	53	≤6
Wood PLA	1.26	3200	39	≤3

**Table 2:** Tensile test results of Clear PLA samples.

Material	Orientation	Sample	Fm	σ_m_	ε_m_	Orientation	Sample	Fm	σ_m_	ε_m_
			[N]	[MPa]	[%]			[N]	[MPa]	[%]
Clear PLA	YZ	1.1 A	2081	51	4.39		1.2 A	1085	32	2.55
		1.1 B	2065	52	4.36		1.2 B	1217	37	2.69
		1.1 C	2060	51	4.34	XY	1.2 C	942	28	2.10
		1.1 D	2066	51	4.37		1.2 D	957	26	2.10
		1.1 E	2055	51	4.34		1.2 E	865	23	1.82

F_m_—maximum force, σ_m_—tensile strength, ε_m_—elongation at tensile strength.

**Table 3:** Tensile test results of White PLA samples.

**Material**	**Orientation**	**Sample**	Fm	σ_m_	ε_m_	**Orientation**	**Sample**	Fm	σ_m_	ε_m_
			[N]	[MPa]	[%]			[N]	[MPa]	[%]
White PLA	YZ	4.1 A	1914	46	4.55		4.2 A	1433	37	4.06
		4.1 B	1947	48	4.56		4.2 B	1212	31	3.21
		4.1 C	1902	43	4.00	XY	4.2 C	1501	38	4.19
		4.1 D	1871	44	3.91		4.2 D	1514	39	4.34
		4.1 E	1708	40	3.66		4.2 E	1450	37	4.25

F_m_—maximum force, σ_m_—tensile strength, ε_m_—elongation at tensile strength.

**Table 4:** Tensile test results of Wood PLA samples.

Material	Orientation	Sample	Fm	σ_m_	ε_m_	Orientation	Sample	Fm	σ_m_	ε_m_
			[N]	[MPa]	[%]			[N]	[MPa]	[%]
Wood PLA	YZ	3.1 A	637	15	3.11		3.2 A	676	17	3.62
		3.1 B	612	15	3.79		3.2 B	693	17	3.94
		3.1 C	666	16	3.54	XY	3.2 C	674	17	3.83
		3.1 D	749	17	3.77		3.2 D	689	17	4.22
		3.1 E	780	19	4.02		3.2 E	703	17	4.44

F_m_—maximum force, σ_m_—tensile strength, ε_m_—elongation at tensile strength.

**Table 5:** Comparison of numerical and experimental tensile test results.

Experiment	F_ma_	σ_ma_	σ_mdev_	ε_ma_	ε_mdev_	Simulation	F_ms_	σ_ms_	ε_ms_	σ_dev_	ε_dev_
	[N]	[MPa]	[MPa]	[%]	[%]		[N]	[MPa]	[%]	[%]	[%]
Clear PLA	2065.4	51.2	0.44	4.4	0.02	Clear PLA	1984	49.6	4.8	−3.12	9.09
White PLA	1868.4	44.2	3.03	4.1	0.40	White PLA	1752	43.8	4.3	−0.90	4.84
Wood PLA	688.8	16.4	1.67	3.6	0.34	Wood PLA	664	16.6	3.7	1.21	2.77

**Table 6:** Comparison of experimental tensile test results with deviations.

Material	Printing Orientation	σ_ma_	σ_mdev_	ε_ma_	ε_mdev_	Printing Orientation	σ_ma_	σ_mdev_	ε_ma_	ε_mdev_
		[MPa]	[MPa]	[%]	[%]		[MPa]	[MPa]	[%]	[%]
Clear PLA	YZ	51.2	0.44	4.4	0.02	XY	29.2	5.45	2.3	0.36
White PLA	YZ	44.2	3.03	4.1	0.40	XY	36.4	3.13	4.0	0.45
Wood PLA	YZ	16.4	1.67	3.6	0.34	XY	17.0	-	4.0	0.32

## Data Availability

Data are contained within the article.

## References

[1] Rajak D.K., Wagh P.H., Linul E. (2022). A Review on Synthetic Fibers for Polymer Matrix Composites: Performance, Failure Modes and Applications. Materials.

[2] Zhang H., Huang T., Jiang Q., He L., Bismarck A., Hu Q. (2021). Recent progress of 3D printed continuous fiber reinforced polymer composites based on fused deposition modeling: A review. J. Mater. Sci..

[3] Valvez S., dos Santos P.S.P., Parente J., Silva M., Reis P. (2020). 3D printed continuous carbon fiber reinforced PLA composites: A short review. Procedia Struct. Integr..

[4] Hikmat M., Rostam S., Ahmed Y.M. (2021). Investigation of tensile property-based Taguchi method of PLA parts fabricated by FDM 3D printing technology. Results Eng..

[5] Hsueh M.-H., Lai C.-J., Wang S.-H., Zeng Y.-S., Hsieh C.-H., Pan C.-Y., Huang W.-C. (2021). Effect of Printing Parameters on the Thermal and Mechanical Properties of 3D-Printed PLA and PETG, Using Fused Deposition Modeling. Polymers.

[6] Abouelmajd M., Bahlaoui A., Arroub I., Zemzami M., Hmina N., Lagache M., Belhouideg S. (2021). Experimental analysis and optimization of mechanical properties of FDM-processed polylactic acid using Taguchi design of experiment. Int. J. Simul. Multidiscip. Des. Optim..

[7] Wang X., Jiang M., Zhou Z.W., Gou J.H., Hui D. (2017). 3D printing of polymer matrix composites: A review and prospective. Compos. Part B Eng..

[8] Pringle A.M., Rudnicki M., Pearce J.M. (2017). Wood Furniture Waste-Based Recycled 3-D Printing Filament. For. Prod. J..

[9] Calì M., Pascoletti G., Gaeta M., Milazzo G., Ambu R. (2020). A New Generation of Bio-Composite Thermoplastic Filaments for a More Sustainable Design of Parts Manufactured by FDM. Appl. Sci..

[10] Sharma V., Roozbahani H., Alizadeh M., Handroos H. (2021). 3D Printing of Plant-Derived Compounds and a Proposed Nozzle Design for the More Effective 3D FDM Printing. IEEE Access.

[11] Gardner D., Anderson J., Tekinalp H., Ozcan S., Sauerbier P. Lignocellulosic-filled polymer feedstocks for large scale additive manufacturing of low cost composites. Proceedings of the International Forest Products Congress.

[12] Velu R., Raspall F., Singamneni S. (2019). Chapter 8-3D printing technologies and composite materials for structural applications. Green Composites for Automotive Applications.

[13] Le Duigou A., Barbé A., Guillou E., Castro M. (2019). 3D printing of continuous flax fibre reinforced biocomposites for structural applications. Mater. Des..

[14] Kariz M., Sernek M., Obućina M., Kuzman M.K. (2018). Effect of wood content in FDM filament on properties of 3D printed parts. Mater. Today Commun..

[15] Tao Y., Wang H., Li Z., Li P., Shi S.Q. (2017). Development and Application of Wood Flour-Filled Polylactic Acid Composite Filament for 3D Printing. Materials.

[16] Spišák E., Nováková-Marcinčínová E., Nováková-Marcinčínová Ľ., Majerníková J., Mulidrán P. (2023). Investigation of the Manufacturing Orientation Impact on the Mechanical Properties of Composite Fiber-Reinforced Polymer Elements in the Fused Filament Fabrication Process. Polymers.

[17] Guessasma S., Belhabib S., Nouri H. (2019). Microstructure and Mechanical Performance of 3D Printed Wood-PLA/PHA Using Fused Deposition Modelling: Effect of Printing Temperature. Polymers.

[18] Živčák J., Nováková-Marcinčínová E., Nováková-Marcinčínová Ľ., Balint T., Puškár M. (2021). Increasing Mechanical Properties of 3D Printed Samples by Direct Metal Laser Sintering Using Heat Treatment Process. J. Mar. Sci. Eng..

[19] Mulidrán P., Spišák E., Tomáš M., Varga J., Majerniková J. (2022). The Effect of Material Models on Springback Prediction. Strength Mater..

[20] Džupon M., Kaščák Ľ., Cmorej D., Čiripová L., Mucha J., Spišák E. (2023). Clinching of High-Strength Steel Sheets with Local Preheating. Appl. Sci..

[21] Nejad R.M., Aliakbari K., Abbasnia S.K., Langari J. (2022). Failure analysis of overdrive gear of passenger car gearbox fabricated from powder metallurgy. Eng. Fail. Anal..

[22] Ezzaraa I., Ayrilmis N., Abouelmajd M., Kuzman M.K., Bahlaoui A., Arroub I., Bengourram J., Lagache M., Belhouideg S. (2023). Numerical Modeling Based on Finite Element Analysis of 3D-Printed Wood-Polylactic Acid Composites: A Comparison with Experimental Data. Forests.

[23] Dutra T.A., Ferreira R.T.L., Resende H.B., Guimarães A. (2019). Mechanical characterization and asymptotic homogenization of 3D-printed continuous carbon fiber-reinforced thermoplastic. J. Braz. Soc. Mech. Sci. Eng..

[24] Park S.-I., Rosen D.W. (2016). Quantifying effects of material extrusion additive manufacturing process on mechanical properties of lattice structures using as-fabricated voxel modeling. Addit. Manuf..

[25] Polyzos E., Van Hemelrijck D., Pyl L. (2021). Numerical modelling of the elastic properties of 3D-printed specimens of thermoplastic matrix reinforced with continuous fibres. Compos. Part B Eng..

[26] Monaldo E., Ricci M., Marfia S. (2023). Mechanical properties of 3D printed polylactic acid elements: Experimental and numerical insights. Mech. Mater..

[27] Chen W., Guo C., Zuo X., Zhao J., Peng Y., Wang Y. (2022). Experimental and Numerical Investigation of 3D Printing PLA Origami Tubes under Quasi-Static Uniaxial Compression. Polymers.

[28] Praveen K., Reddy M., Quoc-Viet P., Prabadevi B., Deepa N., Kapal D., Thippa R.D., Rukhsana R., Madhusanka L. (2022). Industry 5.0: A survey on enabling technologies and potential applications. J. Ind. Inf. Integr..

[29] Pratumpong P., Cholprecha T., Roungpaisan N., Srisawat N., Toommee S., Pechyen C., Parcharoen Y. (2023). Effects of Melt-Blown Processing Conditions on Nonwoven Polylactic Acid and Polybutylene Succinate. Polymers.

[30] Sola A., Trinchi A. (2023). Recycling as a Key Enabler for Sustainable Additive Manufacturing of Polymer Composites: A Critical Perspective on Fused Filament Fabrication. Polymers.

[31] Alrubaie M.A.A., Lopez-Anido R.A., Gardner D.J. (2020). Flexural Creep Behavior of High-Density Polyethylene Lumber and Wood Plastic Composite Lumber Made from Thermally Modified Wood. Polymers.

[32] Carus M., Partanen A. (2018). Natural fibre-reinforced plastics: Establishment and growth in niche markets. JEC Compos. Mag..

[33] (2009). Plastics-Determination of Tensile Properties, Part 1: General Principles.

[34] (2009). Plastics-Determination of Tensile Properties, Part 2: Test Conditions for Plastics for Molding and Extrusion.

[35] Guzanová A., Brezinová J., Varga J., Džupon M., Vojtko M., Janoško E., Viňáš J., Draganovská D., Hašuľ J. (2023). Experimental Study of Steel–Aluminum Joints Made by RSW with Insert Element and Adhesive Bonding. Materials.

[36] Saharudin M.S., Hajnys J., Kozior T., Gogolewski D., Zmarzły P. (2021). Quality of Surface Texture and Mechanical Properties of PLA and PA-Based Material Reinforced with Carbon Fibers Manufactured by FDM and CFF 3D Printing Technologies. Polymers.

[37] Jiang D., Smith D. (2017). Anisotropic mechanical properties of oriented carbon fiber filled polymer composites produced with fused filament fabrication. Addit. Manuf..

[38] Ilyas R.A., Zuhri M.Y.M., Aisyah H.A., Asyraf M.R.M., Hassan S.A., Zainudin E.S., Sapuan S.M., Sharma S., Bangar S.P., Jumaidin R. (2022). Natural Fiber-Reinforced Polylactic Acid, Polylactic Acid Blends and Their Composites for Advanced Applications. Polymers.

[39] Balakrishnan N.K., Siebert S., Richter C., Groten R., Seide G. (2022). Effect of Colorants and Process Parameters on the Properties of Dope-Dyed Polylactic Acid Multifilament Yarns. Polymers.

[40] Bahar A., Hamami A.E.A., Benmahiddine F., Belhabib S., Belarbi R., Guessasma S. (2023). The Thermal and Mechanical Behaviour of Wood-PLA Composites Processed by Additive Manufacturing for Building Insulation. Polymers.

[41] Liu Z., Lei Q., Xing S. (2019). Mechanical characteristics of wood, ceramic, metal and carbon fiber-based PLA composites fabricated by FDM. J. Mater. Res. Technol..

[42] Young R.J. (2001). Polymer Glasses, Mechanical properties of Yielding.

[43] Gleadall A. (2015). Modelling Degradation of Bioresorbable Polymeric Medical Devices. Mechanical Properties of Biodegradable Polymers for Medical Applications.

